# Antimicrobial efficacy of the combination of chlorhexidine digluconate and dexpanthenol

**DOI:** 10.3205/dgkh000284

**Published:** 2016-12-14

**Authors:** Axel Kramer, Ojan Assadian, Torsten Koburger-Janssen

**Affiliations:** 1 Institute of Hygiene and Environmental Medicine, University Medicine Greifswald, Germany; 2 Institute for Skin Integrity and Infection Prevention, University of Huddersfield, UK; 3 Hygiene-North GmbH, Greifswald, Germany

**Keywords:** chlorhexidine digluconate, dexpanthenol, enhancement of microbiocidal efficacy, wound antiseptic

## Abstract

**Objective:** The objective of this standardised experimental study was to investigate the antimicrobial efficacy of the combination of chlorhexidine digluconate (CHX) and the anti-inflammatory pro-vitamin dexpanthenol, which stimulates wound-healing, in the form of Bepanthen^®^ Antiseptic Wound Cream, in order to rule out possible antagonistic combination effects of CHX and the alcohol analogue of pantothenic acid (vitamin B5) dexpanthenol.

**Method:** Testing was carried out using the quantitative suspension test at conditions simulating wound bio-burden. Test strains included *Enterococcus hirae* (ATCC 10541) and *Candida albicans* (ATCC 10231) in accordance with the standard methods of the German Hygiene and Microbiology Society with the following three organic challenges: i) cell culture medium MEM with Earle’s salts, L-glutamine and 10% foetal calf serum (CCM); ii) 10% sheep’s blood; iii) or a mixture of 4.5% albumin, 4.5% sheep’s blood and 1% mucin. For methodological reasons, the wound cream was tested as a 55% dilution, prepared with 1% Tween 80 (equivalent to a content of 0.275% CHX instead of 0.5% as in the original preparation). CHX 0.275% was tested as control in an aqueous solution and in 1% Tween 80. Additionally, 1% Tween 80 was tested in order to rule out an interfering effect of the dilution medium. A combination of 3% Tween 80, 3% saponin, 0.1% histidine, 0.3% lecithin, 0.5% Na-thiosulphate and 1% ether sulphate was identified as the most appropriate neutraliser during the experiments.

**Results:** Exposed to CCM or 10% sheep’s blood, the tested wound cream fulfilled the requirements for a wound antiseptic against both test species with ≥3 log reduction at 10 minutes. Even at the the worst-case challenge test with 4.5% albumin, 4.5% sheep’s blood and 1% mucin, the requirement for a ≥3 log reduction was met after 24 hours of exposure. Interestingly, the aqueous solution of 0.275% CHX tested as control did not achieve the antimicrobial efficacy of the combination of CHX and 5% dexpanthenol. 1% Tween 80 was ineffective against both test species.

**Conclusion:** Bepanthen^®^ Antiseptic Wound Cream achieves the *in vitro* bactericidal and fungicidal efficacy required for a wound antiseptic under three different challenges, despite dilution to 55% of the original preparation. So far, the addition of dexpanthenol was intended to support wound healing. However, our results indicate that the antiseptic efficacy of CHX is synergistically increased by adding 5% dexpanthenol. Acknowledging the antimicrobial and residual efficacy of CHX, and bearing in the mind the contraindications to CHX (allergy and anaphylaxis), the tested wound cream should be regarded as better suitable to be used as wound antiseptic than preparations on basis of CHX alone.

## Zusammenfassung

**Zielsetzung:** In der vorliegenden Untersuchung sollte die mikrobiozide Wirksamkeit der Kombination von Chlorhexidindigluconat (Chx) mit dem antiinflammatorisch wirksamen und die Wundheilung stimulierenden Provitamin Dexpanthenol in Form von Bepanthen^®^ Antiseptische Wundcreme geprüft werden, um mögliche antagonistische Kombinationseffekte von Chx mit dem Analogon der Panthotensäure (Vitamin B5) Dexpanthenol auszuschließen.

**Methode:** Die Testung erfolgte im quantitativen Suspensionstest mit den Teststämmen *Enterococcus hirae* ATCC 10541 und *Candida albicans* ATCC 10231 gemäß den Standardmethoden der Deutschen Gesellschaft für Hygiene und Mikrobiologie mit den drei Belastungen Zellkulturmedium MEM mit Earle-Salzen, L-Glutamin und 10% fetalem Kälberserum (CCM), 10% Schafblut bzw. der Mischung 4,5% Albumin, 4,5% Schafblut und 1% Mucin. Die Wundcreme wurde aus methodischen Gründen als 55%ige Verdünnung, hergestellt mit 1% Tween 80, geprüft (entspricht einem Gehalt von 0,275% Chx anstatt von 0,5% wie im Originalpräparat). Zum Vergleich wurde Chx 0,275% als wässrige Lösung sowie in 1% Tween 80 geprüft. Zusätzlich wurde 1% Tween 80 geprüft, um eine Eigenwirkung auszuschließen. Zur Ausschaltung der mikrobiostatischen Nachwirkung wurde die Kombination 3% Tween 80, 3% Saponin, 0,1% Histidin, 0,3% Lecithin, 0,5% Na-Thiosulfat und 1% Ethersulfat verwendet, die sich in allen Versuchen als am effektivsten zur Neutralisation erwies.

**Ergebnisse:** Bei Belastung sowohl mit CCM als auch mit 10% Schafblut erfüllt die Wundcreme gegen beide Testspecies die Anforderungen an ein Wundantiseptikum mit ≥3 lg innerhalb von 10 min. Bei Testung der worst case-Belastung 4,5% Albumin, 4,5% Schafblut und 1% Mucin wird die Anforderung ≥3 lg innerhalb von 24 h erfüllt.

Die zum Vergleich geprüfte wässrige Lösung von 0,275% Chx erreicht nicht die Wirksamkeit der Wundcreme. Die Wirksamkeit der Lösung von Chx in 1% Tween 80 ist im Vergleich zur wässrigen Lösung noch geringer. Die 1%ige Lösung von Tween 80 war gegen beide Prüforganismen unwirksam.

**Schlussfolgerung:** Bepanthen^®^ Antiseptische Wundcreme erreicht die *in vitro* für ein Wundantiseptikum geforderte bakterizide und levurozide Wirksamkeit bei drei unterschiedlichen Belastungen einschließlich einer worst-case-Situation trotz der Verdünnung auf 55% des Originalpräparats. Die Ergebnisse sprechen dafür, dass durch den Zusatz von 5% Dexpanthenol die antiseptische Wirksamkeit von Chx in vitro erhöht wird. Durch den Zusatz von Dexpanthenol eine Unterstützung der Wundheilung zu erwarten.

Unter Berücksichtigung neuerer Befunde aus dem Schrifttum zur mikrobioziden und remanenten Wirksamkeit von Chx und bei Beachtung der Kontraindikationen für Chx (Allergie und Anaphylaxie) ist die Wundcreme zur Wundantiseptik als geeignet anzusehen.

## Introduction

Pantothenic acid (vitamin B5) and its biologically active precursor D-dexpanthenol (panthenol, pantothenol) activates the proliferation of fibroblasts, promotes the formation of collagen fibres, stimulates the regeneration of damaged tissue, and has an anti-inflammatory and antioxidative effect [1], [2], [3], [4], [5], [6]. D-dexpanthenol has therefore been combined in topical antimicrobial preparations, both antiseptics and antibiotics, intended to be used on wounds [7]. Although the addition of dexpanthenol into antimicrobial wound preparations is aimed to support wound healing, it is not known if the compound may interfere with the antimicrobial action of various antimicrobial compounds. Indeed, an inhibiting effect would particularly have a detrimental effect on antibiotics, as their minimum inhibitory concentration may reach sub-therapeutic dosages. In general, and specifically therefore, the topical application of systemic antibiotics should be opposed, primarily because of the risk of development of bacterial resistance [8], [9]. The WHO also reached the following conclusion: “The use of topical antibiotics and washing wounds with antibiotic solutions are not recommended” [10].

As an alternative to the use of systemic antibiotics in wound antiseptics, therefore, the combination of the antiseptic agent chlorhexidine digluconate (CHX) with dexpanthenol was introduced in the form of Bepanthen^®^ Antiseptic Wound Cream. CHX appears to be relatively safe with little effect on the wound healing process [9]. However, although the use of an antiseptic in a wound healing ointment may be superior to the use of antibiotics, still the question of possible interactions between the two active compounds remains unknown. Therefore, the purpose of the present study was to compare the antimicrobial efficacy of the combination of CHX and dexpanthenol in simulated wound conditions as contained in Bepanthen^®^ Antiseptic Wound Cream, with the efficacy of CHX in an identical concentration dissolved in Tween 80 or dissolved in water, in order to rule out possible interference. 

## Method

Bepanthen^®^ Antiseptic Wound Cream (Bayer Vital GmbH, Leverkusen, Germany) contains 5 mg CHX as the antiseptic active ingredient with the addition of 50 mg dexpanthenol per g which has anti-inflammatory and wound-healing properties. Excipients contained in the formulation are macrogol stearate 1500, glycerol monostearate 40-55, cetomacrogol 1000, liquid paraffin, cetyl stearyl alcohol (Ph.Eur.), dimethicone 1000, glycerol 85%, hard paraffin, hyetellose and purified water. Since the antimicrobial wound cream could not be tested undiluted as an ointment, a 55% dilution was further tested in a quantitative suspension test. The dilution was prepared with 1% Tween 80 (Merck KGaA, Art.-Nr. 8.22187.2500, Lot S6698387). Instead of the original ointment preparation contain 0.5% CHX, the 55% dilution had a CHX content of 0.275%. CHX (Sigma-Aldrich, Product number C9394, Batch BCBK1284V) was tested in an identical concentration to that in the diluted wound cream, i.e. 0.275%, once as an aqueous solution and additionally in 1% Tween 80. The 1% Tween 80 solution used to dilute the cream was also tested alone in order to rule out an interfering effect of the dilution solution.

Testing was carried out using the quantitative suspension test for bactericidal and fungicidal efficacy in accordance with the standard methods of the German Society for Hygiene and Microbiology (DGHM) [11]. A suspension of test organisms in a solution of the interfering substance was mixed with a sample of the test product. *Enterococcus hirae* (ATCC 10541) was used as test bacterium and *Candida albicans* (ATCC 10231) was used to determine the levoricidal efficacy. To simulate wound conditions and wound treatment, exposure times of 10 minutes, 30 minutes, 1 hour, and 24 hours were selected. Three different organic challenges were tested: i) Cell culture medium (CCM) MEM with Earle’s salts, L-glutamine and 10% foetal calf serum; ii) 10% sheep’s blood; and iii) 4.5% albumin, 4.5% sheep’s blood and 1% mucin. 

All tests were performed in duplicate for each contact time and test organism. At the end of the contact time, an aliquot of 0.5 mL was obtained; the microbicidal activity in the sample was immediately neutralized. The number of surviving test organisms in each sample was determined by plating aliquots of the neutralized test suspensions and its dilutions. The reduction was calculated in relation to a control sample containing water instead of the test product (water control, WSH control). The experimental conditions, the non-toxicity of the neutralizer and the dilution-neutralization method were all validated according to the DGHM standard methods: Water control (WSH), method validation (dilution-neutralization method) and non-toxicity of the used neutralizer 3% Tween 80 + 3% saponin + 0.1% histidine + 0.3% lecithin + 0.5% sodium thiosulphate + 1% ether sulphate.

## Results

When exposed to both CCM and 10% sheep’s blood, the tested 55% diluted wound ointment met the requirements for a wound antiseptic against both *E. hirae* and *C. albicans* with ≥3 log within 10 minutes [12]. In the worst-case challenge test with 4.5% albumin, 4.5% sheep’s blood, and 1% mucin, the requirement for a ≥3 log reduction hours was met within 24 hours, which also corresponds to a real-life situation (Table 1 (Tab. 1)). 

The corresponding aqueous solution of 0.275% CHX did not achieve the efficacy of the wound ointment. Antiseptic efficacy against *E. hirae* could not be achieved with exposure to 4.5% albumin + 4.5% sheep’s blood + 1% mucin (Table 1 (Tab. 1)). The efficacy of the solution of CHX in 1% Tween 80 with exposure to 4.5% albumin + 4.5% sheep’s blood + 1% mucin was even lower compared to the aqueous solution (Table 1 (Tab. 1)). As expected, the 1% Tween 80 solution was ineffective against both test organisms.

## Discussion

Our results indicate that the combination of 5% dexpanthenol and CHX showed no antimicrobial antagonisms. Testing was carried out in accordance with the standard method of the German Society for Hygiene and Microbiology for the suspension test [11], however, with some modifications dictated by format of the test composition. Instead of *S. aureus*, *E. faecalis*, and *P. aeruginosa*, *Enterococcus hirae* ATCC 10541 was selected as test strain as this microorganism has proved to be the most resistant compared to the other species tested in parallel [13]. Furthermore, a substantially higher organic challenge was selected as proposed by the DGHM method, which does not cover the condition of the application of antiseptics to wounds. CCM corresponds best to the composition of wound fluid [14]. In order to test the effect of blood, a mixture of 5% albumin, 4.5% sheep’s blood and 1% mucin was used as a further challenge in accordance with the recommendations of Assadian and Kramer [15]. However, it has to be noted that the addition of mucin is not regarded as essential for testing wound antiseptics. Yet, in order to be able to assess efficacy in the case of wounds in the region of the mouth and nasal cavity if necessary, the addition of 1% mucin to 4.5% albumin + 4.5% sheep’s blood was selected as a worst-case scenario in accordance with the recommendations of Pitten et al. [12]. In addition, 10% sheep’s blood was also tested as a challenge in order to take account of the draft Recommendation of the German Society of Hospital Hygiene of 2000 [16]. This took account of the current state of the art in the selection of challenges. Unlike the standard method, only one concentration was tested, as other dilutions were not relevant to actual practice in relation to use as a wound antiseptic. In order to take account of real-life application, exposure times of 10 minutes, 30 minutes, 1 hour and 24 hours were selected. And finally, in order to eliminate a residual bacteriostatic effect of the pathogen suspension inoculated from the test suspension on to the agar, 3% Tween 80 + 3% saponin + 0.1% histidine + 0.3% lecithin + 0.5% sodium thiosulphate + 1% ether sulphate were added to the preparation.

Because of methodological reasons, Bepanthen^®^ Antiseptic Wound Cream had to be tested as a 55% dilution. The addition of 1% Tween 80 to the ointment made it possible to test the preparation as a suspension. Even greater efficacy must therefore be assumed when the wound cream is used in undiluted form. As it can be deduced from the test results for the CHX dilution in 1% Tween 80 that Tween 80 reduces the antimicrobial efficacy of CHX, which is in accordance with previous findings [13]. Therefore, it may be concluded that there is an even greater efficacy reserve for the use of the wound ointment without the test-imposed addition of Tween 80. Whilst efficacy is achieved within 10 minutes without the exposure to mucin, the necessary exposure time was extended to 24 hours with the addition of mucin. Thus, as expected, the addition of mucin had the greatest effect on efficacy. In comparison, the antiseptic active substance polyhexanide loses its antiseptic efficacy in the presence of only 0.5% mucin [17], [18], [19]. In this context, the result with CHX is remarkable. 

The most important finding of the present study is that the antimicrobial efficacy of CHX is increased *in vitro* by adding 5% dexpanthenol. Since the combination achieves an antimicrobial efficacy of ≥3 log during a real-life exposure time – as required for antiseptics – the CHX-based wound cream meets the requirements for a wound antiseptic. However, also other antimicrobial preparations do achieve these criteria in the quantitative suspension test, such as octenidine, polihexanide, PVP-iodine and triclosan. Comparing the antimicrobial efficacy of these antiseptics, CHX is superior to PVP-iodine and triclosan and comparable to the antimicrobial efficacy of octenidine and polihexanide after 24 hours’ exposure time [20]. Since, compared to triclosan and silver compounds, the biocompatibility index is superior [21], CHX may be well suited to be used as a wound antiseptic for this indication. Furthermore, the residual efficacy of CHX is an advantage for a wound antiseptic, which is only exceeded in-vitro by octenidine [22]. However CHX has not been shown to have an allergenic effect in animal experiments. In view of the unusually widespread global use of CHX as an antiseptic, the rare occurrence of allergic reactions [23] should not be overstated.

In some experimental models, mutations have been induced by CHX. The relevance of these findings for humans is unclear. Negative results were obtained in DNA repair assays (umuC, SOS chromotest). The highest dose tested without a mutagenic effect was in the mouse dominant-lethal test 1000 mg/kg/d and in the cytogenetic test in the hamster 250 mg/kg/d [24]. With diploid cells of *A. nidulans*, a test model for the detection of carcinogenic agents, mitotic recombination were induced by 1.5 and 10 µM CHX (in the case of CHX corresponds to 0.0001343% and 0.00089577% respectively) [25]. An increase in chromosomal aberrations in the bone marrow was induced in the mouse following dermal application of 0.2 mL 0.5% CHx solution in distilled water twice daily for 28 days (50 mg/kg) [26]. In human lymphocytes, the micronucleus frequency was significantly increased above 0.5 mg/mL (0.05%): viability was significantly reduced above 0.4 mg/mL [27]. Oral administration for 14 days in the rat induced reversible hyperkeratosis, ulceration and dysplasia in concentrations of 0.2% and to a lesser extent with 0.02% [28]. In the hamster, the only change was an increase in formazan deposition in surface mucosal cell layers [29]. With daily oral administration of 3 mL 0.12% CHX solution for 8 days to rats, there was a significant increase in DNA damage (demonstrated in the comet assay) in leukocytes and kidney cells [30]. In contrast, no local side effects were observed in a 2-year oral study in human subjects [31], [32]. Nor was reproduction toxicity identified. There was no evidence of teratogenicity, embryotoxicity or effects on fertility with 50 mg/kg/d [33], which was confirmed by a more recent study [24] in respect of fertility with up to 100 mg/g/d and in respect of teratogenicity with up to 300 mg/kg/d.

## Conclusion

As Bepanthen^®^ Antiseptic Wound Cream achieves the *in vitro* bactericidal and fungicidal efficacy required for a wound antiseptic with three different organic challenges, including a worst-case scenario. Taking account of recent findings from the literature on the microbicidal and residual effect of CHX, and bearing in mind the contraindications for CHX (allergy and anaphylaxis), the wound ointment may be regarded as suitable for use as a wound antiseptic under real challenging conditions. The microbicidal efficacy is enhanced by the addition of 5% dexpanthenol, which additionally supports wound healing.

## Notes

### Competing interests

The study was initiated and financed by Bayer Vital GmbH, Leverkusen/Germany. Editorial support for this publication was also provided by Bayer Vital GmbH; final responsibility for the English version of this article rests with its authors.

### Funding

No funds were received for this work. 

## Figures and Tables

**Table 1:**
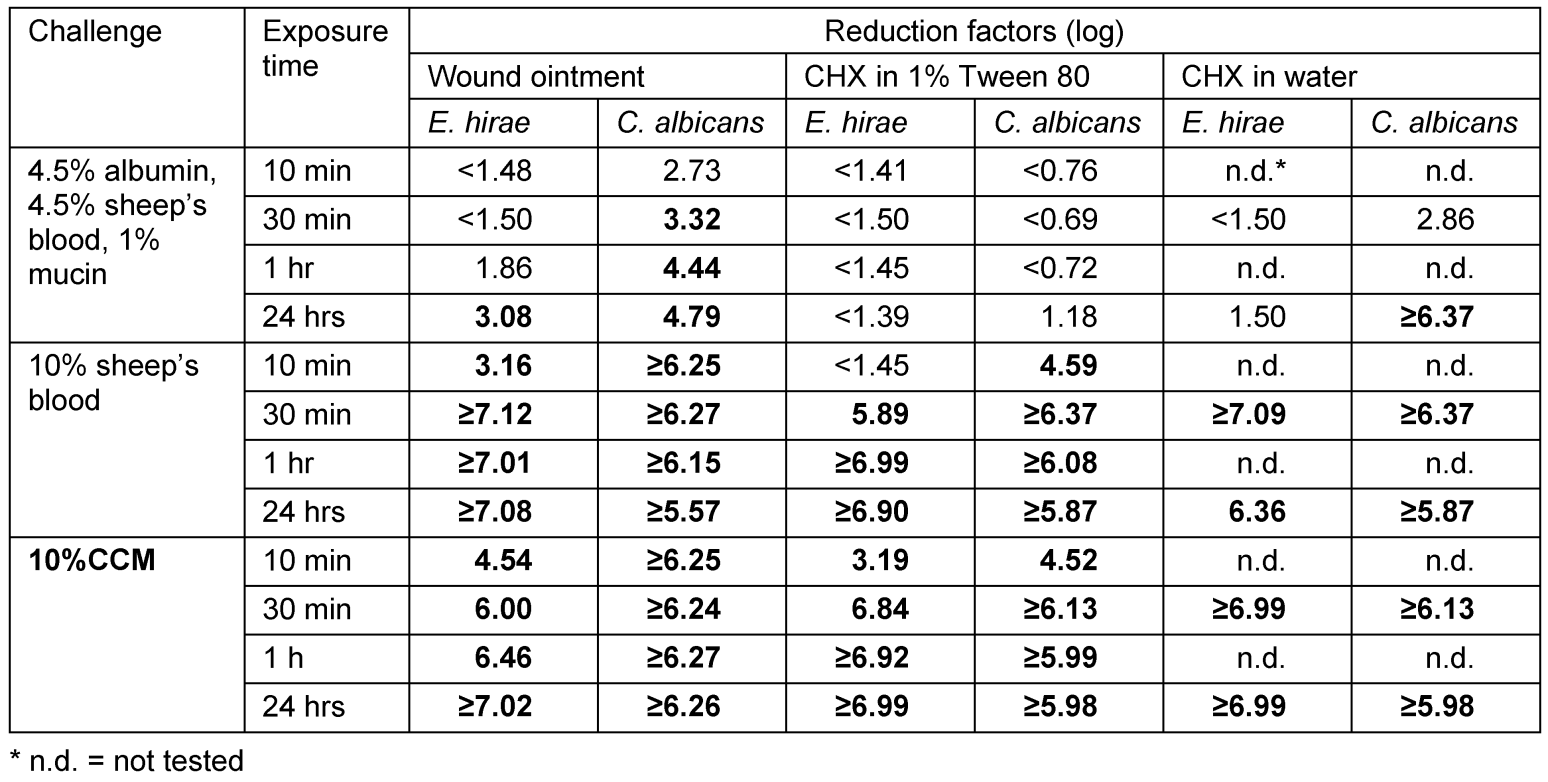
Reduction factors (log) of the bactericidal and fungicidal efficacy of Bepanthen^®^ Antiseptic Wound Cream and the reference/control preparations
